# Complete mitochondrial genome of the freshwater fish *Onychostoma lepturum* (Teleostei, Cyprinidae): genome characterization and phylogenetic analysis

**DOI:** 10.3897/zookeys.1005.57592

**Published:** 2020-12-18

**Authors:** I-Chen Wang, Hung-Du Lin, Chih-Ming Liang, Chi-Chun Huang, Rong-Da Wang, Jin-Quan Yang, Wei-Kuang Wang

**Affiliations:** 1 Department of Environmental Engineering and Science, Feng Chia University, Taichung 407, Taiwan Feng Chia University Taichung Taiwan; 2 The Affiliated School of National Tainan First Senior High School, Tainan 701, Taiwan National Tainan First Senior High School Tainan Taiwan; 3 Taiwan Endemic Species Research Institute, Nantou 552, Taiwan Taiwan Endemic Species Research Institute Nantou Taiwan; 4 Shanghai Universities Key Laboratory of Marine Animal Taxonomy and Evolution, Shanghai Ocean University, Shanghai 201306, China Shanghai Ocean University Shanghai China

**Keywords:** Cyprinid, mitogenome, *Onychostoma lepturum*, phylogeny, population genetics, Southeast Asia

## Abstract

The cyprinid genus *Onychostoma* Günther, 1896 consists of 24 valid species distributed in Southeast Asia, including Taiwan, Hainan, mainland China and the Indochina region. In the present study, we determined the complete mitochondrial genome of *O. lepturum*, which is 16,598 bp in length, containing 13 protein-coding genes, two rRNA genes, 22 tRNA genes and a typical control region (D-loop). To verify the molecular phylogeny of the subfamily Acrossocheilinae, we provide new insights to better understand the taxonomic status of *Acrossocheilus*, *Onychostoma* and *Folifer brevifilis*. The phylogenetic trees presented three major clades based on the 13 protein-coding genes from 28 Acrossocheilinae species. Clades I and II represent the *Onychostoma* and *Acrossocheilus* groups, respectively. Species of *Acrossocheilus*, *Onychostoma* and *F. brevifilis* are included in Clade III, which is considered as an ancestral group. This work provides genomic variation information and improves our understanding of the Acrossocheilinae mitogenome, which will be most valuable in providing new insights for phylogenetic analysis and population genetics research.

## Introduction

The cyprinid genus *Onychostoma* Günther, 1896 consists of 24 valid species distributed in Southeast Asia, including Taiwan, Hainan, mainland China and the Indochina region (Song et al. 2018; Froese 2019). *Onychostoma* is composed of mountain stream cyprinids that are characterized primarily by the possession of a sharp, cornified sheath cutting edge in the lower jaw and no fleshy lower lip (Shan et al. 2000). Among these species, four species are only distributed in the Indochina region, including Laos Vietnam and Thailand, eleven species are endemic to mainland China, one species is endemic to Taiwan island, and the remaining seven species are shared with mainland China and the Indochina region (Froese 2019). *Onychostoma lepturum* (Boulenger, 1900) is distributed in Laos and Vietnam and can also be found in the Yuanjiang River in mainland China and Hainan Island (Shan et al. 2000; Kottelat 2001).

The Cyprinidae family has the most species of any freshwater fish family. The family encompasses 11 subfamilies, with the genus *Onychostoma* belonging to the Acrossocheilinae subfamily (Yang et al. 2015). The taxonomic placement of the Asian genus *Onychostoma* has undergone many changes. *Onychostoma* was synonymized with the African genus *Varicorhinus* (e.g., Taki 1975), but recent studies demonstrate that *Onychostoma* and *Varicorhinus* fall in two distinct major clades in terms of chromosome numbers and molecular markers (Yang et al. 2015). According to previous studies, *Acrossocheilus* and *Onychostoma* were reported to be paraphyletic or polyphyletic (Yang et al. 2015; Wang et al. 2016; Zheng et al. 2016). Among these were Acrossocheilini, *Acrossocheilus*, *Onychostoma* and *Folifer brevifilis* (Peters, 1881), which formed a clade in the molecular analysis (Yang et al. 2015; Wang et al. 2016; Zheng et al. 2016). Three genera (*Acrossocheilus*, *Onychostoma* and *Folifer*) have been previously placed in different taxa together with many other cyprinines (Chen et al. 1984; Yue 2000). However, the phylogenetic relationships of these three genera, based on different molecular markers, has been controversial.

*Folifer brevifilis* is closely related to *O. simum* (Sauvage & Dabry de Thiersant, 1874) based on the mitochondrial and nuclear markers (Yang et al. 2015; Wang et al. 2016; Zheng et al. 2016). According to mitochondrial genomes, *O. simum* and *O. gerlachi* (Peters, 1881) were in sister groups (Zhang et al. 2018). The genus *Acrossocheilus* represents three separate lineages: the barred species (e.g., *A. beijiangensis* (Wu & Lin, 1977) and *A. iridescens* (Nichols & Pope, 1927)), non-barred species (e.g., *A. yunnanensis* (Regan, 1904)) and *A. monticola* (Günther, 1888) based on mitochondrial DNA sequences (Zheng et al. 2016). However, Hou et al. (2020) reassigned species of the *Acrossocheilus* cluster into two separate clades: Clade I (an ancestral clade), including *A. monticola* and *A. yunnanensis*, and Clade II, which was further divided into two sublineages (subclades A and B) based on available whole mitochondrial genomes. Subclade B clustered with *A. longipinnis* (Wu, 1939), *A. iridescens* and *A. barbodon* (Nichols & Pope, 1927), and subclade A included other *Acrossocheilus* species (e.g., *A. parallens* (Nichols, 1931), *A. hemispinus* (Nichols, 1925), *A. jishouensis* (Zhao, Chen & Li, 1997) (Hou et al. 2020). Yang et al. (2015) and Zheng et al. (2016) proposed that members of *Onychostoma* be divided into three groups based on morphological and molecular data. Zhang et al. (2018) also showed that the eight species of *Onychostoma* cluster into three separate lineages based on the whole mitochondrial genome sequence. However, similar research in Zhai et al. (2020) only identified two lineages among nine species of *Onychostoma* with the same molecular markers. Interestingly, Zhang et al. (2018) proposed that *O. rarum* (Lin, 1933) was the sister group of *O. alticorpus* (Oshima, 1920), but recently another study showed that *O. rarum* seems to be more closely related to *O. barbatulum* (Pellegrin, 1908) and *O. barbatum* (Lin, 1931) (Zhai et al. 2020). In the previous studies, the classification of the three groups was inconsistent (Yang et al. 2015; Zheng et al. 2016; Zhang et al. 2018). For example, *O. lepturum* (Boulenger, 1900) clustered together with *O. meridionale* (Kottelat, 1998) (Yang et al. 2015). In addition, Wang et al. (2016) and Zheng et al. (2016) proposed that *O. lepturum* was nested with *O. gerlachi*.

Recent population analyses suggested that the nucleotide diversity of cyprinids on Hainan Island was lower than that of cyprinids in mainland China (Zhou et al. 2017). A decline in freshwater fish resources has been observed due to the effects of overfishing, water pollution and environmental deterioration in China (Kang et al. 2013, 2014). The phylogenetic relationships among species in the genera *Onychostoma* and *Acrossocheilus* have been studied based on morphological data and on nuclear and mitochondrial genes (Wang et al. 2007; Xin 2008; Wang et al. 2016; Zheng et al. 2016). However, the current understanding among members of the genera *Onychostoma* and *Acrossocheilus* and their internal phylogenetic relationships remains confusing. The complete mitochondrial genome has alternative molecular markers for phylogenetic analysis capable of providing much more robust phylogenetic reconstructions than smaller portions of the mtDNA (Huang et al. 2017; Hou et al. 2020). Mitogenomes are thought to be reliable markers for reconstructing phylogenies in recent taxonomic and phylogenetic studies of cyprinids (Huang et al. 2017; Chung et al. 2020).

Previous studies suggest an inclusive phylogenetic clade including species from *Acrossocheilus*, *Onychostoma*, and *Folifer brevifilis* based on molecular markers (Yang et al. 2015; Zheng et al. 2016). Although a previous study has characterized the complete mitochondrial genome of *O. lepturum* (Zhai et al. 2020), the genome annotation, comparative analysis and the phylogenetic relationships of *Onychostoma* remain poorly understood due to the limited genomic data used. Our approach better informs the conservation of this species; thus, we determined the complete mitochondrial genome of *O. lepturum* based on next-generation sequencing data and assessed its phylogenetic relationships with another 11 available mitogenomes in the genus *Onychostoma* and 16 available mitogenomes in the genus *Acrossocheilus* and *F. brevifilis*, with an available mitogenome in *Spinibarbus hollandi* (Oshima, 1919) used as an outgroup.

## Materials and methods

### Sample and DNA extraction

The sample of *Onychostoma lepturum* was caught from the Lingshui River in Baoting County of Hainan in China (18°42'07"N, 109°40'44"E). Samples were collected from the field sites with seines, fatally anesthetized with MS-222 (Sigma, St. Louis, MO) and fixed and stored in 100% ethanol. All specimens are lodged in the laboratory of Jin-Quan Yang, Shanghai Ocean University, Key Laboratory of Exploration and Utilization of Aquatic Genetic Resources. All animal experiments were carried out in accordance with the guidelines and with approval of the Animal Research and Ethics Committee of Shanghai Ocean University (permissions, SHOU-DW-2018-021). Total genomic DNA was extracted from muscle tissue using the Genomic DNA Purification Kit (Gentra Systems, Valencia, CA) in the laboratory.

### Sequencing and genome annotation and analysis

The complete mitogenome of *O. lepturum* was obtained from high-throughput sequencing of whole-genome DNA with a HiSeqX Ten platform (Illumina, San Diego, CA) with a paired-end 150 bp approach. Next-generation sequencing (NGS) was used to perform low-coverage whole-genome sequencing to obtain the complete mitogenome according to a previous protocol (Chiu et al. 2018). By using commercial software (Geneious V9, Auckland, New Zealand), approximately 1.1% of raw reads (34,001 out of 29,140,518) were assembled de novo to produce a single, circular form of the complete mitogenome with an average coverage of 245 X. Compared to the corresponding complete mtDNA sequence of genus *Onychostoma*, 13 protein-coding genes and ribosomal RNA (rRNA) genes were identified using Clustal X 1.83 (http://www.clustal.org/). Codon usage, nucleotide substitution and base composition were determined using MEGA X (Kumar et al. 2018), and the skewing of the nucleotide composition was measured in terms of AT- and GC-skews according to the following formulas: AT-skew = (A – T)/(A + T) and GC-skew = (G – C)/(G + C) (Perna and Kocher 1995).

### Phylogenetic analysis

Phylogenetic analyses using a total of 11 mitogenomes of *Onychostoma* species, 16 mitogenomes of *Acrossocheilus* species and one mitogenome of *F. brevifilis* were performed based on Neighbor-joining (NJ), Maximum-likelihood (ML), and Bayesian (BI) methods, with *Spinibarbus hollandi* as the outgroup. Twenty-nine mitogenomes were downloaded from NCBI and were aligned using MEGA X (alignment with CLUSTALW) with default settings (Kumar et al. 2018). The best model GTR +G (General Time Reversible model with Gamma distributed rates among sites) was chosen based on the Akaike information criterion (AIC) using the smart model selection algorithm (Lefort et al. 2017), and the ML trees were constructed using PhyML 3.0 software (Guindon et al. 2010). The statistical confidences were assessed through the bootstrap test inferred from 1000 replicates. A NJ tree was constructed based on the Kimura 2-parameter model with 1000 bootstrap replicates using MEGA X (Kumar et al. 2018). The Bayesian inference (BI) tree was conducted using the GTR+G model strategy with MrBayes 3.2.6 (Ronquist and Huelsenbeck 2003), and two independent Markov Chains Monte Carlo (MCMC) chains were run for 5 × 10^7^ generations; the first 50,000 trees before stationarity were discarded as burn-in, and the remaining trees were used to construct the majority-rule consensus trees. Effective sample size (ESS) values, as computed by plotting the log likelihood scores against the generation times using the program Tracer 1.7 (Rambaut et al. 2018), were above 200 for the convergence of MCMC runs.

## Results

### Mitochondrial genomic structure and composition

**Figure 1.**
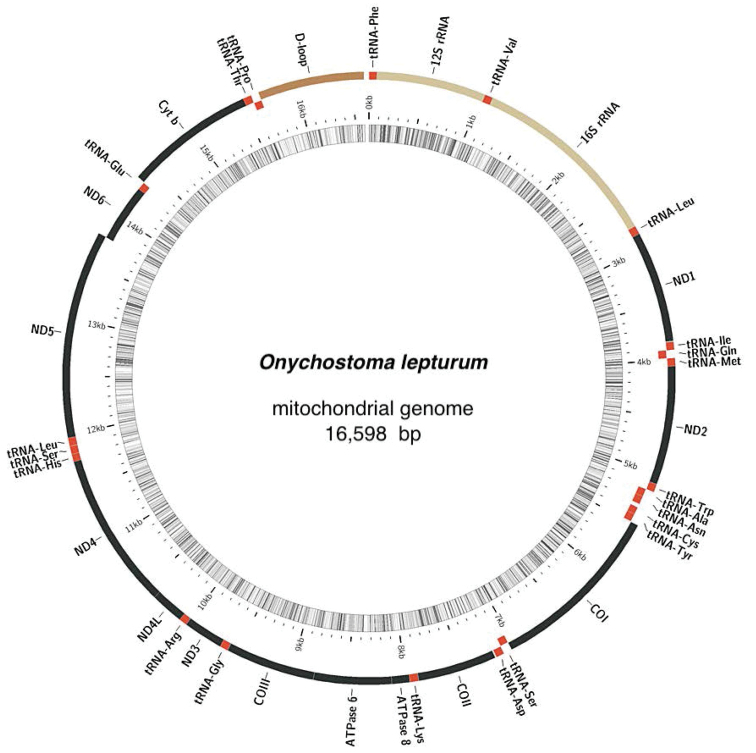
Gene map of the mitochondrial genome of *Onychostoma lepturum*. Two rRNA genes (in red); 13 coding genes (in green); 22 tRNA genes and control region (D-loop) (in yellow). (Color figure online).

In the present study, the complete mitochondrial genome sequence of *O. lepturum* derived by NGS was found to be 16,598 bp and was deposited in GenBank (accession MT258556). The mitogenome contained 37 typical mitochondrial genes with 13 typical vertebrate protein-coding genes, 2 ribosomal RNA (rRNA) genes, 22 tRNAs, and a control region (D-Loop) (Fig. 1). Most of the *O. lepturum* mitochondrial genes were encoded on the H-strand, although the ND6 and eight tRNA genes (tRNA^Gln^, tRNA^Ala^, tRNA^Asn^, tRNA^Cys^, tRNA^Tyr^, tRNA^Ser^, tRNA^Glu^ and tRNA^Pro^) were encoded on the L-strand (Fig. 1). The total length was found to be similar to that of the other *Onychostoma* sequences compared, differing from them by between 1 and 9 bp. The mitogenome base composition is 31.3% A, 16.2% G, 24.0% T, and 28.6% C, with a slight AT bias (55.3%). Eleven of thirteen protein-coding genes in *O. lepturum* started with a typical ATG codon, except for the COI and ATP6 genes, which were GTG. Seven protein-coding genes ended with the termination codon TAA (ND1, CO1, ATP8, ATP6, ND4L, ND5, and ND6), while the remaining six genes terminated with a single base T (Table 1). The 22 tRNA genes ranged in size from 67 to 76 bp, and the length of tRNA^Cys^ gene (67 bp) was the shortest, whereas the longest were the tRNA^Leu^ and tRNA^Lys^ genes (76 bp). The noncoding control region (D-loop) is located between tRNA^Phe^ and tRNA^Pro^ and is 937 bp in length (Table 1). The genes in the *O. lepturum* mitogenome were closely arranged with overlapping and interval phenomena. There is a total of 22 bp in overlaps between six gene junctions, and each single overlap ranged in size from 1 to 7 bp, with the longest overlapping region (7 bp) located between ATP8/ATP6 and ND4L/ND4, 4 bp of overlapping regions between ND5 and ND6 and fewer than 2 bp at the remaining three positions (Table 1). However, there are 11 intergenic spacer regions ranging in size from 1 to 35 bp (67 bp in total), and the largest spacer (35 bp) is located between tRNA^Asn^ and tRNA^Cys^ (Table 1).

**Table 1.** Organization of the mitochondrial genome of *Onychostoma lepturum*.

Locus	Position			Codon				
	start	stop	size(bp)	start	stop	anti-codon	intergenic nucleotide^*^	strand^+^
tRNA^Phe^	1	69	69			GAA	0	H
12s rRNA	70	1030	961				0	H
tRNA^Val^	1031	1102	72			TAC	0	H
16s rRNA	1103	2783	1681				0	H
tRNA^Leu^	2784	2859	76			TAA	0	H
*ND1*	2860	3834	975	ATG	TAA		0	H
tRNA^lle^	3840	3911	72			GAT	4	H
tRNA^Gln^	3910	3980	71			TTG	-2	L
tRNA^Met^	3983	4051	69			CAT	2	H
*ND2*	4052	5096	1045	ATG	T–		0	H
tRNA^Trp^	5097	5168	72			TCA	0	H
tRNA^Ala^	5171	5239	69			TGC	2	L
tRNA^Asn^	5241	5313	73			GTT	1	L
tRNA^Cys^	5349	5415	67			GCA	35	L
tRNA^Tyr^	5415	5486	72			GTA	-1	L
*COI*	5488	7038	1551	GTG	TAA		1	H
tRNA^Ser^	7039	7109	71			TGA	0	L
tRNA^Asp^	7113	7184	72			GTC	3	H
*COII*	7197	7887	691	ATG	T–		12	H
tRNA^Lys^	7888	7963	76			TTT	0	H
*ATP8*	7965	8129	165	ATG	TAG		1	H
*ATP6*	8123	8805	683	GTG	TA-		-7	H
*COIII*	8806	9590	785	ATG	TAA		0	H
tRNA^Gly^	9591	9662	72			TCC	0	H
*ND3*	9663	10011	349	ATG	T–		0	H
tRNA^Arg^	10012	10081	70			TCG	0	H
*ND4L*	10082	10378	297	ATG	TAA		0	H
*ND4*	10372	11752	1381	ATG	T–		-7	H
tRNA^His^	11753	11821	69			GTG	0	H
tRNA^Ser^	11822	11890	69			GCT	0	H
tRNA^Leu^	11892	11964	73			TAG	1	H
*ND5*	11965	13788	1824	ATG	TAA		0	H
*ND6*	13785	14306	522	ATG	TAG		-4	L
tRNA^Glu^	14307	14375	69			TTC	0	L
*Cytb*	14381	15521	1141	ATG	T–		5	H
tRNA^Thr^	15522	15594	73			TGT	0	H
tRNA^Pro^	15594	15663	70			TGG	-1	L
D-loop	15664	16598	935				0	H

**Figure 2.**
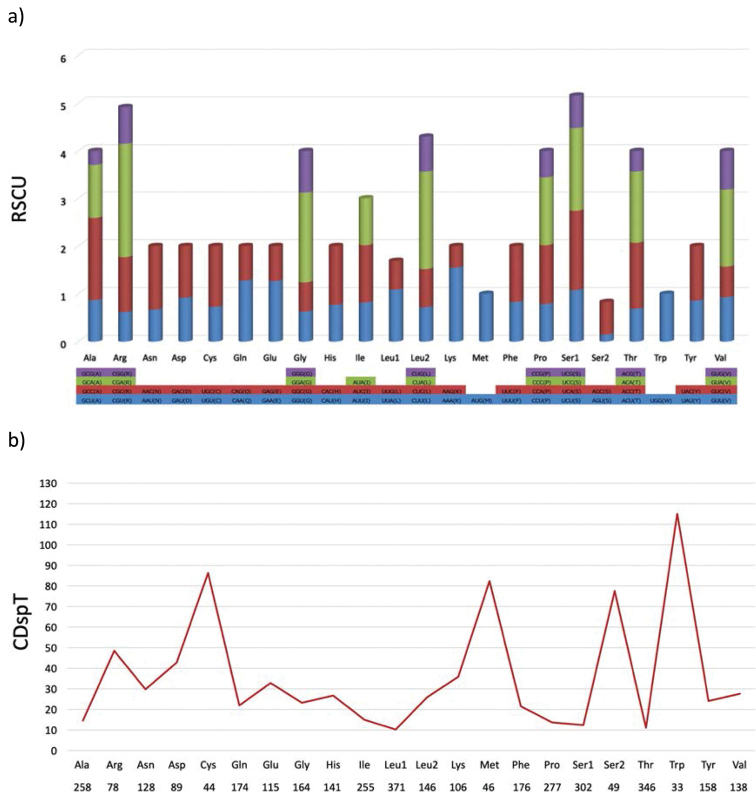
Comparison of codon usage in mitochondrial genomes of *Onychostoma lepturum***a** Relative synonymous codon usage (RSCU) in the *Onychostoma lepturum* mitogenome. Codon families are provided on the X-axis, and the RSCU values, on the Y-axis **b** Codon distribution in the *Onychostoma lepturum* mitogenome. CDspT, codons per thousand codons. Codon families are provided on the X-axis.

We assessed the amino acid (AA) codon usage by calculating the relative synonymous codon usage (RSCU) values in 13 PCGs, which are shown in Fig. 2a. A total of 3803 codons were encoded by 13 PCGs, and the most frequently used codons were CUA (4.7%), ACA (3.4%) and ACC (3.1%). In the PCGs of the *O. lepturum* mitogenome, the AA components and their codon usage reveal that one codon family (Trp) represents more than 100 codons per thousand codons (CDpT), three codon families (Cys, Met and Ser2) between 50 CDpT and 100 CDpT, and the other twenty codon families less than 50 CDpT (Fig. 2b).

Comparative analysis of nucleotide base composition showed that the composition of *O. lepturum* is identical to that of the other *Onychostoma* fishes, and most of the genes within these *Onychostoma* species maintain a consistent position and direction (Table 2). The analysis of our complete dataset nucleotide frequencies confirmed a bias for A, showing averages of 31.3% of A, 16.2% G, 24.0% T, and 28.6% C, with a slight AT bias (55.3%), which is similar to the patterns found in most fish mitogenomes. The PCGs have a slightly higher AT content (54.7%) than ribosomal RNA genes (53.6%) (Table 2). Furthermore, the A+T content at codon site 2 of PCGs (58.7%) was slightly higher than that at site 3 (58.5%), while the A+T content at the first site was the lowest (47.1%). The A+T-rich region was the main noncoding region (control region) of mitogenomes, has the highest A+T content (66.5%), which was significantly higher than other genes of the mitogenome and was typical of animal mitochondrial genomes (Zhang and Hewitt 1997; Zhou et al. 2017). The AT and GC skews of *O. lepturum* are 0.132 and -0.277, respectively (Table 2). The GC-skew of all genes was negative and revealed a similar pattern of base composition behavior for the *O. lepturum* mitogenome, except for tRNA which was positive (0.049) (Table 2). Strand asymmetry in nucleotide composition is usually reflected by AT and GC skews, which is a remarkable feature of animal mitochondrial genomes (Wei et al. 2010). The mitogenome contents of 11 *Onychostoma* species were calculated and showed an A+T bias, ranging from 55.3% (*O. lepturum*) to 56.6% (*O. barbatulum*) (Suppl. material 1: Table S1). The analysis of *Onychostoma* mitochondria populations showed distinct skew patterns, in which AT was positive and GC was negative (Suppl. material 1: Table S1).

**Table 2.** Nucleotide composition of the *Onychostoma lepturum* mitochondrial genome.

	Length (bp)	T%	C%	A%	G%	A+T%	AT-skew%	GC-skew%
Genome	16598	24	28.6	31.3	16.2	55.3	0.132	0.277
PCGs	11409	25.7	29.4	29	15.8	54.7	0.06	-0.3
1^st^ codon position	3807	20.3	27	26.8	25.8	47.1	0.138	-0.023
2^nd^ codon position	3802	40.1	27.7	18.6	13.6	58.7	-0.366	-0.341
3^rd^ codon position	3800	16.8	33.6	41.7	8	58.5	0.423	-0.615
rRNA	2642	19.1	25.8	34.5	20.6	53.6	0.287	-0.112
tRNA	1566	26.6	21.5	28.2	23.7	54.8	0.029	0.049
D-loop	935	32.5	20.3	34	13.2	66.5	0.022	-0.212

The 22 tRNA genes in the *O. lepturum* mitogenome are interspersed between rRNA and protein-coding genes, with sizes ranging from 67 to 76 bp; tRNA^Cys^ was the shortest (67 bp), while tRNA^Leu^ and tRNA^Lys^ were the longest (76 bp). The arrangement of 8 L-strand encoded and 14 H-strand-encoded tRNA genes is similar to the distributions observed in other *Onychostoma* species. Two rRNA genes were identified on the L-strand in *O. lepturum*, which is similar to the other *Onychostoma* species, with a total length of 2642 bp. The 16S rRNA is located between tRNA^Val^ and tRNA^Leu^, with a length of 1681 bp, whereas the 12S rRNA is located between tRNA^Phe^ and tRNA^Val^, with a length of 961 bp. Regarding the two rRNA genes, the GC-skew is slightly negative (–0.112), but the AT-skew is strongly positive (0.287). The total A+T content of the rRNA genes (53.1%) is lower than those of the total tRNA genes (55.0%) and the total PCG genes (55.6%).

### Phylogenetic analyses

**Figure 3.**
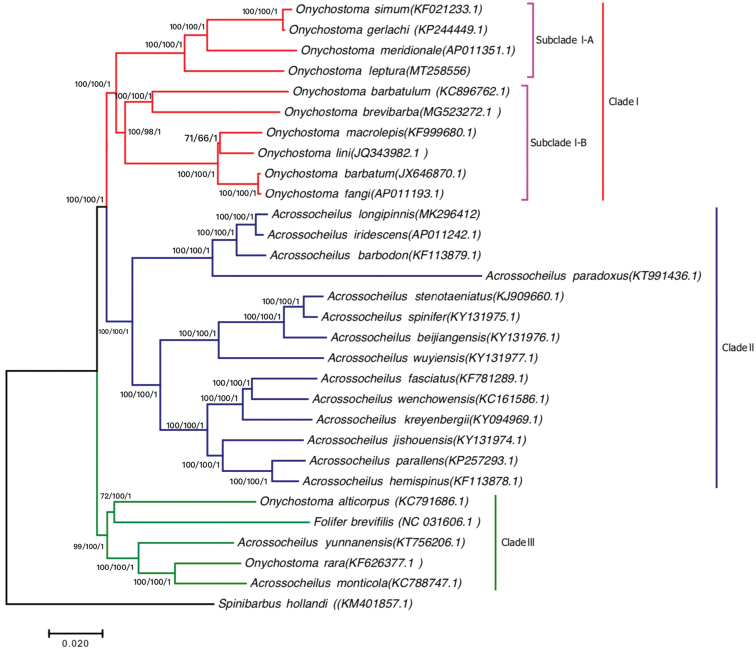
Phylogenetic trees derived from Maximum-Likelihood (ML) and Neighbor Joining (NJ) approaches based on whole mitochondrial genomes. The numbers on the nodes are the bootstrap values of ML/NJ. The number after the species name is the GenBank Accession Number.

To further investigate the phylogenetic position of *O. lepturum* within the genera *Acrossocheilus* and *Onychostoma*, the concatenated set of nucleotide sequences of available whole mitochondrial genomes from 10 *Onychostoma* species, 16 *Acrossocheilus* species and *F. brevifilis* were derived for phylogenetic reconstruction, along with *S. hollandi* as an outgroup. The ML, NJ and BI analyses showed the same topology, representing the three main lineages (Clades I, II and III). The phylogenetic tree revealed that Clade I is the *Onychostoma* group, which can be separated into two subclades (subclades I-A and I-B) with strong support (Fig. 3). The phylogenetic position of *O. lepturum* is closer to that of *O. meridionale* than to that of *O. simum* and *O. gerlachi* in subclade I-A. Subclade I-B clusters with five species of *Onychostoma*, including *O. barbatulum*, *O. barbatum*, *O. fangi*, *O. lini* and *O. macrolepis*. Clade II is the *Acrossocheilus* group and includes 14 species, which is in accordance with traditional classifications based on morphology and previous phylogenetic studies based on whole mitochondrial genomes (Yuan et al. 2015; Hou et al. 2020). Species of *Acrossocheilus*, *Onychostoma* and *F. brevifilis* are included in Clade III, which is considered as an ancestral group, including *A. monticola*, *A. yunnanensis*, *O. rarum*, *O. alticorpus* and *F. brevifilis*.

## Discussion

### Mitochondrial genome of *O. lepturum*

The total length of the *Onychostoma* mitogenomes ranged between 16,590 (*O. rarum*) and 16,601 bp (*O. simum* and *O. gerlachi*), while that of the *O. lepturum* is a typical closed circular DNA molecule with a length of 16,598 bp (Fig. 1; GenBank No. MK296412), making it similar to other *Onychostoma* sequences, which differ by between 3 and 8 bp (16,590 bp for *O. rarum* and 16,601 bp for *O. simum*, *O. gerlachi*) (Suppl. material 1: Table S1). We suggest that the variability observed in closely related mitogenome length can be caused by variations in tandem repeat elements within the control region (D-Loop); differences in the lengths of intergenic regions are also a likely explanation for the gene overlaps (Fig. 1). The nucleotide composition of the *O. lepturum* mitogenome is highly biased toward A+T (55.3%), which is similar to the values in other *Onychostoma* species and displays strand skewness consistent with asymmetrical mutation pressure (Bielawski and Gold 2002) (Suppl. material 1: Table S1). Of the 22 identified tRNAs, the AT and GC skews were both positive in the *O. lepturum* mitogenome, which is common in *Onychostoma* mitogenomes. Comparative mitogenomic structure, organization and gene arrangement analyses of all *Onychostoma* mitogenomes are conserved without any structural rearrangement. Among *Onychostoma* fishes that also exhibited highly similar nucleotide compositions and codon usage patterns, a slight difference was also observed in some species. Ten of the thirteen total PCGs used ATG as the initiation codon (ND1, ND2, ND4, ND4L, ND5, ND6, COII, COIII, ATP8, and Cyt *b*), whereas the COI gene started with the GTG codon in all *Onychostoma* species. Most protein-coding genes started with a traditional ATG codon except for COI in *Onychostoma* mitogenomes, which is consistent with previous reports for other fish mitogenomes (Satoh et al. 2016). In the ATP6 gene, only *O. lepturum* was found with GTG as the initiation codon, and the other *Onychostoma* species used the typical ATG codon. In contrast, only ND3 held diverse start codons in *Onychostoma* mitogenomes. Five *Onychostoma* species were initiated by GTG, including *O. alticorpus*, *O. rarum*, *O. simum*, *O. gerlachi* and *O. meridionale*. The other *Onychostoma* species was initiated by ATG (Table 3). In addition, six PCGs (ND1, COI, ATP8, ND4L, ND5 and ND6) harbored the typical termination codons (TAA and TAG) in the *Onychostoma* mitochondrial genome, and the remaining PCGs were terminated with incomplete codons (T- and TA-). The incomplete termination codon T in the *Onychostoma* mitochondrial genome was a common termination codon (ND2, ND3, ND4, COII and Cyt *b*), except for *O. alticorpus*, which was terminated by TAA in the ND3 gene (Table 3); however, the COI, ND4L and ND5 genes used TAA, the ATP8 gene used TAG, while ND1 and ND6 used TAG or TAA (Table 3). ND1 and ND6 are terminated with the codon TAA for most *Onychostoma* species, which is different from the termination codon (TAG) observed for ND1 in *O. barbatulum*, *O. simum* and *O. gerlachi* and ND6 in *O. lepturum*, *O. barbatulum*, *O. alticorpus*, *O. simum*, *O. gerlachi* and *O. meridionale* (Table 3). We suggest that ND1 and ND6 appear to have evolved relatively rapidly in these *Onychostoma* species, and a similar observation has also been reported in a previous study of *Acrossocheilus* mitogenomes (Hou et al. 2020).

**Table 3.** Composition and skewness in mitogenomes of 12 *Onychostoma* species.

Species	ND1	ND2	COI	COII	ATP8	ATP6	COIII	ND3	ND4L	ND4	ND5	ND6	Cytb	GenBank
*O. lepturum*	ATG/TAA	ATG/T–	GTG/TAA	ATG/T–	ATG/TAG	GTG/TA-	ATG/TA-	ATG/T–	ATG/TAA	ATG/T–	ATG/TAA	ATG/TAG	ATG/T–	MT258556
*O. macrolepis*	ATG/TAA	ATG/T–	GTG/TAA	ATG/T–	ATG/TAG	ATG/TA-	ATG/TA-	ATG/T–	ATG/TAA	ATG/T–	ATG/TAA	ATG/TAA	ATG/T–	KF999680.1
*O. lini*	ATG/TAA	ATG/T–	GTG/TAA	ATG/T–	ATG/TAG	ATG/TA-	ATG/TA-	ATG/T–	ATG/TAA	ATG/T–	ATG/TAA	ATG/TAA	ATG/T–	JQ343982.1
*O. barbatum*	ATG/TAA	ATG/T–	GTG/TAA	ATG/T–	ATG/TAG	ATG/TA-	ATG/TA-	ATG/T–	ATG/TAA	ATG/T–	ATG/TAA	ATG/TAA	ATG/T–	JX646870.1
*O. fangi*	ATG/TAA	ATG/T–	GTG/TAA	ATG/T–	ATG/TAG	ATG/TA-	ATG/TA-	ATG/T–	ATG/TAA	ATG/T–	ATG/TAA	ATG/TAA	ATG/T–	AP011193.1
*O. barbatulum*	ATG/TAG	ATG/T–	GTG/TAA	ATG/T–	ATG/TAG	ATG/TA-	ATG/TA-	ATG/T–	ATG/TAA	ATG/T–	ATG/TAA	ATG/TAG	ATG/T–	KC896762.1
*O. alticorpus*	ATG/TAA	ATG/T–	GTG/TAA	ATG/T–	ATG/TAG	ATG/TA-	ATG/TA-	GTG/TAA	ATG/TAA	ATG/T–	ATG/TAA	ATG/TAG	ATG/T–	KC791686.1
*O. rarum*	ATG/TAA	ATG/T–	GTG/TAA	ATG/T–	ATG/TAG	ATG/TA-	ATG/TA-	GTG/T–	ATG/TAA	ATG/T–	ATG/TAA	ATG/TAA	ATG/T–	KF626377.1
*O. simum*	ATG/TAG	ATG/T–	GTG/TAA	ATG/T–	ATG/TAG	ATG/TA-	ATG/TA-	GTG/T–	ATG/TAA	ATG/T–	ATG/TAA	ATG/TAG	ATG/T–	KF021233.1
*O. gerlachi*	ATG/TAG	ATG/T–	GTG/TAA	ATG/T–	ATG/TAG	ATG/TA-	ATG/TA-	GTG/T–	ATG/TAA	ATG/T–	ATG/TAA	ATG/TAG	ATG/T–	KP244449.1
*O. meridionale*	ATG/TAA	ATG/T–	GTG/TAA	ATG/T–	ATG/TAG	ATG/TA-	ATG/TA-	GTG/T–	ATG/TAA	ATG/T–	ATG/TAA	ATG/TAG	ATG/T–	AP011351.1
*O. brevibarba*	ATG/TAA	ATG/T–	GTG/TAA	ATG/T–	ATG/TAG	ATG/TA-	ATG/TA-	ATG/T–	ATG/TAA	ATG/T–	ATG/TAA	ATG/TAG	ATG/T–	MG523272.1

### Molecular phylogeny of *Onychostoma*

Taki (1975) suggested that two groups were subgenera of the genus *Onychostoma*: *Onychostoma* and *Gymnostomus*. These two groups are divided based on their possession of osseous simple dorsal rays; the subgenus Gymnostomus includes those having non-osseous rays, while the subgenus Onychostoma includes those with osseous simple dorsal rays (Taki 1975). There are five species (*O. lepturum*, *O. barbatulum*, *O. barbatum*, *O. macrolepis* and *O. alticorpus*) belonging to the subgenus Gymnostomus, whereas the remaining species belongs to the subgenus Onychostoma. According to the mouth width, mouth-opening shape, and post-labial groove length, previous studies divided members of *Onychostoma* into three groups (Chen 1989; Xin et al. 2009). Our results showed that the *Onychostoma* with osseous simple dorsal rays (*O. simum*, *O. rara*, *O. lini*, *O. meridionale*, *O. gerlachi* and *O. fangi*) could not be successfully clustered together (Fig. 3). In addition, molecular evidence revealed that Clade I, including subclades I-A (*O. lepturum*, *O. meridionale*, *O. simum* and *O. gerlachi*) and subclade I-B (*O. barbatulum*, *O. barbatum*, *O. fangi*, *O. lini* and *O. macrolepis*), comprise a stable monophyletic group.

Comparison was made between the phylogenetic trees constructed by Yang et al. (2015), Wang et al. (2016) and Zheng et al. (2016), whereby input sequences such as mtDNA and nuclear sequences were used for phylogeny. One of the similarities between all studies is that Clade I in the *Onychostoma* group comprises a stable monophyletic group distinct from *Acrossocheilus* and *F. brevifilis*. Following our study, *O. simum* was consistently grouped closely with *O. gerlachi* in the *Onychostoma* group (Clade I-A), as in the previous study based on the mitogenome data (Zhang et al. 2018). However, one distinct difference among these above mentioned studies is that in the trees constructed by Yang et al. (2015), Wang et al. (2016) and Zheng et al. (2016) based on nuclear and mitochondrial genes, *O. simum* was found to be more closely related to *O. alticorpus* and *F. brevifilis*. *Onychostoma simum* was more closely related to *O. gerlachi* and was distributed in the Lancang Jiang and Red River basins (Song et al. 2018), as described in a previous study based on the biogeography of *Onychostoma* (Zhang et al. 2018). The resulting relationships are not consistent with earlier conclusions based on morphological characteristics (Taki 1975; Xin et al. 2009) or on nuclear and mitochondrial genes (Wang et al. 2007; Yang et al. 2016; Zheng et al. 2016). However, the single-gene evolutionary tree of the COI gene is inconsistent with that of combined complete mitochondrial genome, suggesting that *O. simum* (KF021233.1) were incorrectly identified. According to NCBI data and Wang et al. (2013), specimens KF021233.1 did not have an associated collection site (and we suggest that specimens KF021233.1 were *O. gerlachi*). Our results show that *O. lepturum* was found to be more closely related to *O. meridionale* than *O. gerlachi*. *Onychostoma lepturum* and *O. meridionale* occur in Laos and Vietnam, while *O. lepturum* is distributed in the Yuanjiang River in mainland China and Hainan Island. According to the previous biogeographic studies, the Gulf of Tonkin once formed part of the coastal plain of mainland China and the River in Vietnam and Hainan Island drained into the Gulf of Tonkin during Pleistocene glaciations (Zhang et al. 2020).

Recent studies have revealed similar scenarios in the genetic patterns of *Garra orientalis* (Yang et al. 2016), *Aphyocypris normalis* (Huang et al. 2019) and *Opsariichthys hainanensis* (Zhang et al. 2020). Xin et al. (2009) proposed that *O. lini* and *O. barbata* were found to be related closer to *O. macrolepis* based on morphological characters. This result suggested that the phylogenetic relationship of subclade I-B are concordant with the molecular phylogenetic and morphological analyses. In addition, Clade II is the *Acrossocheilus* group, which includes 14 species, comprising a stable monophyletic group distinct from the *Onychostoma* group in the ML, NJ and BI trees (Fig. 3). The present study strongly supported that some species of *Acrossocheilus*, *Onychostoma* and *F. brevifilis* belong to the monophyletic group (Clade III), including *A. monticola*, *A. yunnanensis*, *O. rarum*, *O. alticorpus* and *F. brevifilis*. Interestingly, our results corroborate the previous finding that *O. rarum* is the sister group of *O. alticorpus* (Hoang et al. 2015; Song et al. 2018; Zhang et al. 2018), but Zhai et al. (2020) suggested that *O. rarum* appeared to be more closely related with *O. barbatulum* and *O. barbatum*. We suggested that the specimens of *O. rarum* (NC022869.1) were also misidentified. Moreover, all of these members were previously confirmed as a monophyletic group (Yuan et al. 2015; Hou et al. 2020). Overall, the complete mtDNA sequence of *O. lepturum* provides useful genetic data for addressing further questions in the systematics and evolutionary history of *Onychostoma*, for understanding its molecular diversity and for genetic conservation applications.

## Supplementary materials

Supplementary material 1 Table S1 Data type DNA Explanation note Composition and skewness in the genus *Onychostoma* mitogenomes. https://binary.pensoft.net/file/488501This dataset is made available under the Open Database License (http://opendatacommons.org/licenses/odbl/1.0/). The Open Database License (ODbL) is a license agreement intended to allow users to freely share, modify, and use this Dataset while maintaining this same freedom for others, provided that the original source and author(s) are credited.I-Chen Wang, Hung-Du Lin, Chih-Ming Liang, Chi-Chun Huang, Rong-Da Wang, Jin-Quan Yang, Wei-Kuang Wang
